# Effects of different radical distal gastrectomy on postoperative inflammatory response and nutritional status in patients with gastric cancer

**DOI:** 10.3389/fsurg.2023.1112473

**Published:** 2023-03-15

**Authors:** Xuefei Cheng, Chuandong Wang, Yi Liu, Xiaojuan Zhang, Liyuan Zhou, Zhizun Lin, Wei Zeng, Lihang Liu, Changshun Yang, Weihua Li

**Affiliations:** ^1^ Shengli Clinical Medical College, Fujian Medical University, Fuzhou, China; ^2^ Department of Surgical Oncology, Fujian Provincial Hospital, Fuzhou, China; ^3^ Department of Endoscopy, National Cancer Center/Cancer Hospital, Chinese Academy of Medical Science and Peking Union Medical College, Beijing, China; ^4^ Fuzong Clinical Medical College, Fujian Medical University, Fuzhou, China

**Keywords:** gastric cancer, total laparoscopic surgery, inflammation, nutritional status, neutrophil/Lymphocyte ratio, prognostic nutrition index

## Abstract

**Objectives:**

The inflammatory response caused by gastric cancer surgery and the low nutritional status of patients with gastric cancer can cause growth of tumour cells, reduce immunity, and increase tumour burden. We investigated the effects of different surgical methods on postoperative inflammatory response and nutritional status in patients with distal gastric cancer.

**Methods:**

Clinical data of 249 patients who underwent radical distal gastrectomy for distal gastric cancer from February 2014 to April 2017 were retrospectively analysed. Patients were divided according to the surgical method (open distal gastrectomy [ODG], laparoscopic-assisted distal gastrectomy [LADG] and total laparoscopic distal gastrectomy [TLDG]). Characteristics of different surgical procedures, including inflammation parameters and nutritional indicators, and different time points (preoperatively, 1 day postoperatively, and 1 week postoperatively) were compared using non-parametric test analysis.

**Results:**

At postoperative day 1, white blood cell count [WBC], neutrophil count [N], neutrophil/lymphocyte ratio [NLR], and platelet/lymphocyte ratio [PLR] increased in the three groups, and ΔN and ΔNLR were significant; the smallest change was observed in TLDG (*P* < 0.05). Albumin [A]and prognostic nutrition index [PNI] significantly decreased; the smallest ΔA and ΔPNI, which were statistically significant, were noted in TLDG. One week postoperatively, WBC, N, NLR, and PLR decreased, and WBC, N, and NLR showed significant difference. A and PNI of the three groups increased after 1 week, and A and PNI showed significant differences.

**Conclusion:**

Postoperative inflammatory response and nutritional status of patients with distal gastric cancer are associated with the surgical technique. TLDG has little influence on the inflammatory response and nutritional level compared with LADG and ODG.

## Introduction

With the development of minimally invasive techniques, total laparoscopic radical distal gastrectomy is currently one of the surgical techniques for distal gastric cancer. Although its application is increasing, its clinical value remains controversial. Several studies showed that the size of the surgical incision is related to local inflammatory response. Both open and minimally invasive surgeries have certain influence on the overall inflammatory response of the body (1), but the specific mechanism is unclear. Some scholars reported that mononuclear cell and cytokine levels after laparoscopic surgery are lower than those after open surgery (2–7) Additionally, postoperative patients with gastric cancer are prone to malnutrition. Patients with gastric cancer who had different radical surgeries have different postoperative levels of nutritional indicators (albumin, prognostic nutrition index, etc.). Low nutritional status among postoperative patients with gastric cancer may inhibit the body's humoral immunity and cellular immune function, thereby reducing the body's immunity to tumours and thus leading to tumour recurrence.

Hence, this study aimed to assess the relationship between different surgical techniques (open distal gastrectomy [ODG], laparoscopic-assisted distal gastrectomy [LADG] and total laparoscopic distal gastrectomy [TLDG]) and the body's inflammatory response and nutritional status based on the inflammatory markers (white blood cell count [WBC], neutrophil count [N], neutrophil/lymphocyte ratio [NLR] and platelet/lymphocyte ratio [PLR]) and the nutritional indicators (albumin [A]and prognostic nutrition index [PNI]).

## Material and methods

### Study design

In this retrospective study, standard demographic and clinicopathological data of 503 patients with distal gastric cancer who underwent radical distal gastrectomy from February 2014 to December 2017 in Fujian Provincial Hospital were obtained. All patients were diagnosed by gastroscopy and pathological examination before operation. Inclusion criteria were pathologically confirmed gastric cancer with TNM stages I, II and III; radical resection through distal gastrectomy; no liver, lung or other distant organ metastasis and no abdominal implant transfer; no major heart or lung dysfunctions. Exclusion criteria included perioperative complications (Clavien-Dindo grade II or higher), such as anastomotic leakage, arterial embolization, postoperative bleeding and gastric motility complications; palliative or emergency surgery; perioperative infection; history of blood transfusion, active bleeding or bleeding disorders in the past 2 months; and immunosuppressive therapy. After applying the exclusion criteria, the clinical data of 249 patients were retrospectively analysed. Patients were divided according to the different surgical techniques (ODG, LADG and TLDG). The effects of the different surgical methods on the body were evaluated and compared using inflammatory and nutritional indicators preoperatively, 1 day postoperatively and 1 week postoperatively. This study was reviewed and approved by the Ethics Committee of Fujian Provincial Hospital. Data were anonymized, and the requirement for informed consent from the patients was waived. All study procedures were performed in accordance with the Helsinki Declaration of 1964 and later versions.

### Surgical procedure

**Figure 1:**
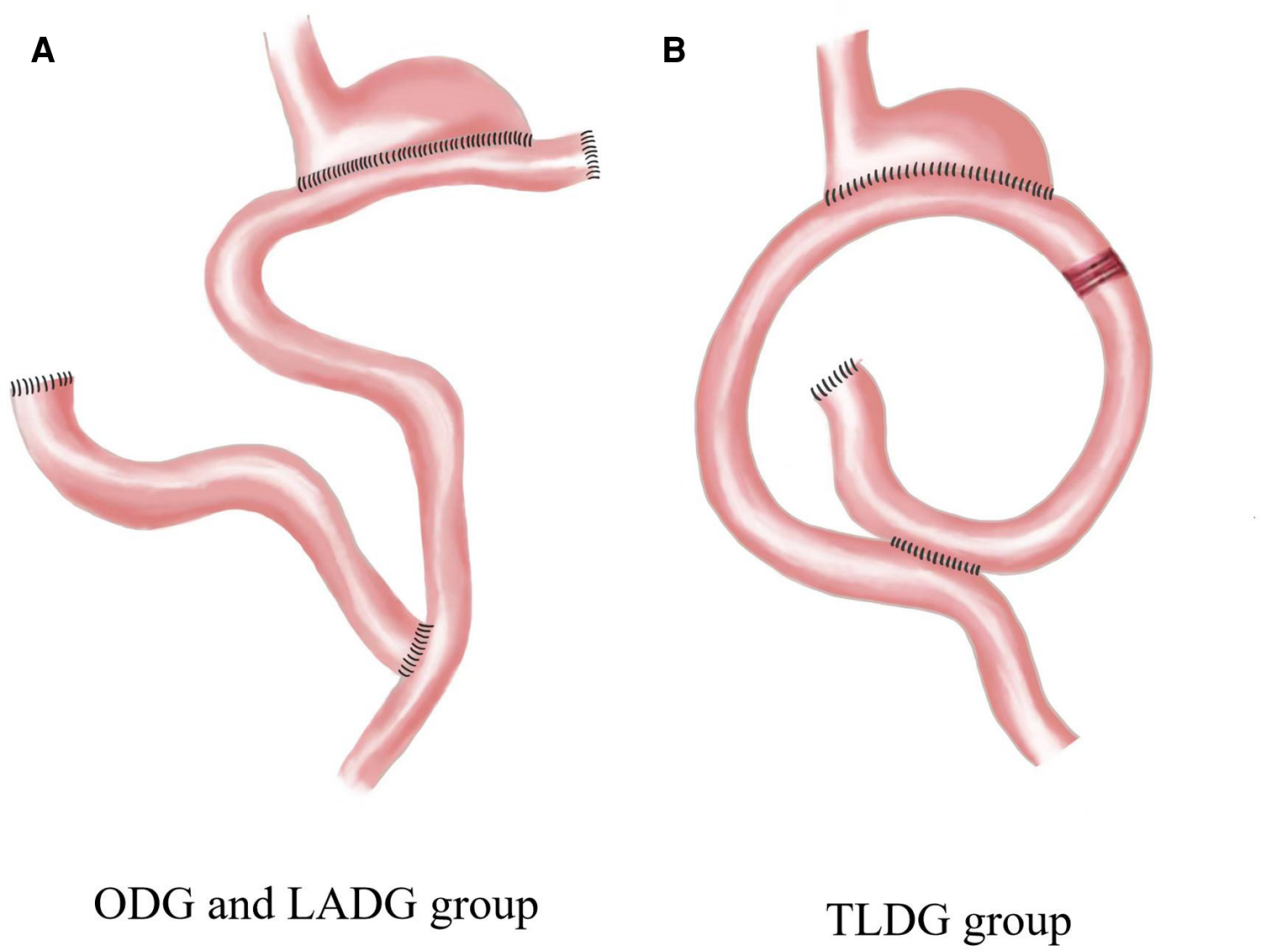
Gastrointestinal reconstruction. ODG, open distal gastrectomy; LADG, laparoscopic-assisted distal gastrectomy; TLDG, total laparoscopic distal gastrectomy.

General anaesthesia was induced *via* tracheal intubation. The most distal part of the stomach was resected according to the classic method (8), and the D2 lymphadenectomy was performed according to the 14th edition of the Japanese Gastric Cancer Treatment Protocol. The gastrointestinal reconstruction performed in the three groups differed, with the ODG and LADG groups undergoing proximal residual stomach-jejunum Roux-en-Y anastomosis and the TLDG group undergoing proximal residual stomach-jejunum uncut Roux-en-Y anastomosis (Figure 1).

### Indicators

Routine blood and biochemical examinations were performed at 8 am preoperatively, 1 day postoperatively and 1 week postoperatively. WBC, N, L, PLT and A were recorded. The NLR and PLR were determined. The WBC, N, NLR and PLR were evaluated as the inflammatory parameters. Changes in the NLR (ΔNLR), PLR (ΔPLR), WBC (ΔWBC) and N (ΔN) during the perioperative period were evaluated to assess the body's inflammatory response. Moreover, PNI was calculated as follows: PNI = A [g/L] + 5 × L [×10^9^/L] (9). ΔPNI and ΔA were calculated at different time points to determine the level of nutrition. The inflammatory response and nutritional status of the patients were evaluated based on the aforementioned indicators preoperatively, 1 day postoperatively and 1 week postoperatively.

### Statistical analysis

Data were analysed using chi-squared test tests or Fisher's exact test to compare proportions. Non-parametric analysis of variance (Kruskal-Wallis method) was employed in the intra- and inter-group evaluations. Differences with *P* values < 0.05 were considered statistically significant. All statistical analyses were performed using SPSS version 20.0 (IBM Corp., Armonk, NY, USA). Pictures were drawn with GraphPad Prism version 7 (GraphPad Software, San Diego, CA, USA).

## Results

### Baseline data

No significant differences in the baseline data including sex, age, T stage, N stage, TNM stage, Borrmann type, pathological type and preoperative comorbidities were found among the three surgical methods; however, tumour size and operation time showed statistically significant difference (Table 1). Preoperatively, no significant differences in the markers WBC, N, NLR, PLR, A and PNI were observed among the three surgical methods.

**Table 1:** Clinicopathological characteristics.

Characteristics	ODG(*n* = 85)	LADG(*n* = 99)	TDLG(*n* = 65)	*Χ*^2^orF	*P*
Sex					
Male	58	68	45	0.170	0.992
Female	27	31	20		
Age (years)	58.28 ± 12.36	58.66 ± 10.78	58.23 ± 9.91	0.155	0.925
Surgery Time (min)	205.68 ± 51.24	271.82 ± 57.00	231.14 ± 44.53	65.326	0.000
Tumor size (cm)	3.83 ± 1.71	3.17 ± 1.86	3.61 ± 2.40	10.890	0.004
T-Stage					
1	21	31	20	7.026	0.318
2	15	18	10		
3	15	12	17		
4	34	37	18		
N-Stage					
0	33	49	37	7.920	0.244
1	13	16	12		
2	17	13	6		
3	22	21	10		
TNM					
I	24	39	27	7.630	0.106
II	22	22	21		
III	39	38	17		
Pathology type					
Adenocarcinoma	64	76	51	0.250	0.993
Signet-ring cell carcinoma	6	6	4		
Other cancer^b^	15	17	10		
Differentiated					
Well	31	35	25	3.775	0.437
Median	11	23	13		
Poor^a^	43	41	27		
Borrmann type					
0	3	13	2	10.486	0.106
1	8	8	6		
2	25	33	18		
3	49	45	39		

^a^ Poor: poor differentiated and undifferentiated.

^b^ Other cancer: the mixed cancer of stomach cancer, intramucosal carcinoma, neuroendocrine carcinoma, medullary carcinoma, epithelioid carcinoma and adenosquamous carcinoma.

### Inflammation indicators

At 1 day postoperatively, the WBC, N, NLR and PLR increased compared with their preoperative values. N and NLR in ODG, LADG and TLDG showed statistically significant difference (*P* = 0.000 and *P* = 0.002, respectively); however, PLR did not show significant statistical difference (Table 2). ΔN (*P* = 0.001) and *Δ*NLR (*P* = 0.006) showed significant statistical difference. LADG and TLDG were statistically different between ΔN (*P* = 0.014) and ΔNLR (*P* = 0.020). No statistically significant difference in ΔWBC and ΔPLR was noted among the three groups (Table 3).

**Table 2:** Characteristics with different surgeries at different times.

A Characteristics with different surgical of preoperative					
Characteristics	ODG (*n* = 85)	LADG (*n* = 99)	TDLG (*n* = 68)	H	*P*
WBC(×10^9^/L)	6.05 ± 1.85	6.20 ± 2.06	6.03 ± 1.52	0.070	0.966
N(×10^9^/L)	3.69 ± 1.68	3.71 ± 1.85	3.50 ± 1.17	0.419	0.811
A(g/L)	41.34 ± 5.45	42.65 ± 4.39	42.62 ± 3.44	4.323	0.115
NLR(N/L)	2.42 ± 1.91	2.30 ± 2.54	1.99 ± 0.94	2.306	0.316
PLR(PLT/L)	158.85 ± 83.29	146.56 ± 60.15	148.36 ± 77.09	0.465	0.793
PNI[5*L(*10^9^/L)+A(g/L)]	50.25 ± 7.08	51.98 ± 5.62	52.12 ± 5.02	4.361	0.113
B Characteristics with different surgical of postoperative 1 day					
Characteristics	ODG (*n* = 85)	LADG (*n* = 99)	TDLG (*n* = 68)	H	*P*
WBC(×10^9^/L)	12.78 ± 4.47	12.20 ± 3.92	11.67 ± 3.05	2.246	0.325
N(×10^9^/L)	11.53 ± 3.99	10.57 ± 3.65	8.96 ± 2.26	16.874	0.000
A(g/L)	27.48 ± 5.67	30.39 ± 3.87	32.26 ± 3.45	36.118	0.000
NLR(N/L)	15.87 ± 8.32	14.88 ± 8.96	11.42 ± 4.86	12.725	0.002
PLR(PLT/L)	270.28 ± 114.59	268.56 ± 124.88	255.47 ± 112.27	0.709	0.701
PNI[5*L(*10^9^/L)+A(g/L)]	31.64 ± 5.88	34.56 ± 4.30	36.65 ± 4.15	33.565	0.000
C Characteristics with different surgical of postoperative 1 week					
Characteristics	ODG (*n* = 85)	LADG (*n* = 99)	TDLG (*n* = 65)	H	*P*
WBC(×10^9^/L)	9.81 ± 2.57	9.01 ± 2.96	7.30 ± 2.02	36.757	0.000
N(×10^9^/L)	7.01 ± 2.34	6.55 ± 2.56	5.28 ± 1.51	26.404	0.000
A(g/L)	34.09 ± 4.34	35.14 ± 3.73	35.97 ± 3.45	8.319	0.016
NLR(N/L)	6.69 ± 3.41	6.09 ± 4.43	5.15 ± 2.79	10.005	0.007
PLR(PLT/L)	233.64 ± 116.88	227.02 ± 122.03	214.22 ± 110.58	1.063	0.588
PNI[5*L(*10^9^/L)+A(g/L)]	40.24 ± 5.79	41.38 ± 4.81	42.28 ± 5.09	7.633	0.022

WBC, White blood cell count;, N, Neutrophils count; L, Lymphocytes count; PLT, Platelets count; A, Albumin.

NLR, Neutrophil-to-lymphocyte ratio; PLR, Platelet-to-lymphocyte ratio; PNI, 5* lymphocyte (*10^9^/L)+ albumin (g/L),.

**Table 3:** Comparison of change of indicators before surgery to postoperative 1 day.

Characteristics	ODG (*n* = 85)	LADG (*n* = 99)	TLDG (*n* = 65)	*P*_1_	*P*_2_	*P*_3_	*P*_4_
ΔWBC	6.73 ± 4.73	6.00 ± 3.53	5.64 ± 3.01	0.308	0.218	0.914	0.431
ΔN	7.01 ± 2.34	6.49 ± 2.56	5.46 ± 2.37	0.198	0.000	0.014	0.001
ΔA	13.86 ± 6.51	12.25 ± 4.59	10.35 ± 3.97	0.152	0.001	0.003	0.001
ΔNLR	13.45 ± 8.50	12.58 ± 9.55	9.43 ± 4.85	0.330	0.002	0.020	0.006
ΔPLR	111.42 ± 107.01	121.99 ± 123.55	107.12 ± 100.04	0.819	0.802	0.699	0.917
ΔPNI	18.61 ± 7.78	17.42 ± 5.49	15.46 ± 5.13	0.280	0.006	0.014	0.009

*P*1: *P* value after comparison between ODG and LADG.

*P*2: *P* value after comparison between ODG and TLDG.

*P*3: *P* value after comparison between LADG and TLDG.

*P*4: Compared ODG, LADG and TLDG after the *P* value.

At 1 week postoperatively, the WBC, N, NLR and PLR decreased but remained higher than their preoperative values. A significant difference in WBC (*P* = 0.000), N (*P* = 0.000) and NLR (Table 2C; *P* = 0.007) was observed among ODG, LADG and TLDG, but no significant difference in PLR was noted. The ΔWBC (2.97 ± 4.98 vs. 3.19 ± 3.88 vs. 4.37 ± 2.87; *P* = 0.021) was statistically significant. The difference between the two groups was that the ODG and TLDG could be statistically different from ΔWBC (2.97 ± 4.98vs. 4.37 ± 2.87; *P* = 0.012). The LADG and TLDG with regards to ΔWBC (3.19 ± 3.88vs. 4.37 ± 2.87; *P* = 0.020) was statistical difference. There were no significant statistical differences between ΔN, ΔNLR and ΔPLR in the three groups (Table 4).

**Table 4:** Comparison of change of indicators postoperative 1 day to postoperative 1 week.

Characteristics	ODG (*n* = 85)	LADG (*n* = 99)	TLDG (*n* = 65)	*P*_1_	*P*_2_	*P*_3_	*P*_4_
ΔWBC	2.97 ± 4.98	3.19 ± 3.88	4.37 ± 2.87	0.508	0.012	0.020	0.021
ΔN	3.98 ± 2.92	4.71 ± 3.93	3.34 ± 3.99	0.438	0.343	0.720	0.585
ΔA	6.61 ± 6.71	4.75 ± 4.92	3.7 ± 4.86	0.066	0.004	0.108	0.011
ΔNLR	9.18 ± 7.92	8.78 ± 9.53	6.28 ± 5.24	0.757	0.048	0.085	0.110
ΔPLR	36.64 ± 135.76	41.54 ± 168.40	41.25 ± 123.08	0.858	0.786	0.712	0.924
ΔPNI	8.60 ± 7.42	6.82 ± 5.49	5.62 ± 6.26	0.165	0.006	0.046	0.014

*P*1: *P* value after comparison between ODG and LADG.

*P*2: *P* value after comparison between ODG and TLDG.

*P*3: *P* value after comparison between LADG and TLDG.

*P*4: Compared ODG, LADG and TLDG after the *P* value.

### Nutritional markers

At 1 day postoperatively, A and PNI decreased compared with the preoperative values. The differences in A (*P* = 0.000) and PNI (*P* = 0.000)(Table 2B), ΔA (*P* = 0.001) and ΔPNI (*P* = 0.009) among ODG, LADG and TLDG were statistically significant (Table 3). *Δ*A (*P* = 0.001) and ΔPNI (*P* = 0.006) increased in ODG and TLDG. A statistically significant difference in ΔA (*P* = 0.003) and ΔPNI (*P* = 0.014) was observed between LADG and TLDG (Table 3).

At 1 week postoperatively, A and PNI increased but remained lower than their values at 1 week preoperatively. The differences in A (*P* = 0.016) and PNI (*P* = 0.022) were statistically significant (Table 2C). The increase in the amplitude of ODG and TLDG there was significant at *Δ*A (6.61 ± 6.71 vs. 3.70 ± 4.86; *P* = 0.004) and ΔPNI (8.60 ± 7.42 vs. 5.62 ± 6.26; *P* = 0.006), LADG and TLDG was compared regarding ΔPNI (6.82 ± 5.49vs. 5.62 ± 6.26; *P* = 0.046) and was statistically significant. (Table 4).

## Discussion

Comparison of the preoperative, 1-day postoperative and 1-week postoperative values of the inflammation index and nutritional indicators among TLDG, ODG and LADG showed that the different surgical methods cause different levels of inflammation and nutrition, with TLDG causing the least trauma to the body.

We observed a minimum change in ΔNLR and ΔN preoperatively to 1 day postoperatively in TLDG, which indicates that TLDG has the weakest level of inflammatory response to the body. Moreover, the intensity of the postoperative inflammatory response can be determined primarily based on WBC and N; however, there are some disadvantages that the degree of surgery affects WBC and N to a greater extent. Therefore, we mainly used NLR, which more objectively reflects the level of inflammation of the body in different surgical methods, combined with the trend of WBC and N and the range of changes. Therefore, the intensity of inflammatory response in patients with radical distal gastric cancer is related to the type of surgery. Studies have shown that the extent of postoperative immune response is associated with surgically induced wounds. Immune response could induce systemic or local inflammation in the body, which in turn impairs the immune function of the body and increases the susceptibility to infectious complications (10–12). In a large meta-analysis with a large sample, Y. Jiang et al. (13) found that PLR is associated with low survival in patients with metastatic and non-metastatic solid tumours. Previous studies showed that the higher the NLR value in gastric cancer patients, the shorter the survival rate and overall survival time (12–14). However, we also observed from the results that the *Δ*PLR as an indicator of inflammation was not statistically significant, but it was consistent with the trend of inflammatory response changes, whereas the amount of TLDG was minimal. We suspect that patients with advanced gastric cancer have tumour cell growth that consumes platelets and that traumatic platelet consumption is associated with it. In addition, tumour-associated platelets secrete *5*-hydroxytryptamine, platelet factor 4, tumour growth factor *β* and other particles, which adhere to vascular damage, thus maintaining the integrity of the tumour vascular endothelium and promoting the progression of tumour cells (15).

From another perspective, the three surgical methods differ. Incision size, which has the most direct effect on the body, differs among the three surgical methods: the ODG incision is approximately 15 cm on average (largest); the LADG incision is about 8–12 cm; the TLDG incision is approximately 3–5 cm (smallest). The size of the incision during surgery is related to the extent of the inflammatory response and could induce the production and release of inflammatory mediators near the incision. In addition, studies have shown that inflammation due to wounds could increase the proliferation of mesothelial cells and increase the number of inflammatory cells (16) The inflammatory response to surgery stimulates the body to form cellular immunity; the infiltration of concentrated granulocytes, macrophages and myofibroblasts stimulates the release of a large amount of inflammatory mediators and cellular chemokines (17, 18). More interestingly, Krall et al. (1) established a standard experimental model of surgery and wound healing response and showed that distant metastasis linked tumour cell growth and wound healing cascade and that the recruitment of neutrophils and macrophages is followed by infiltration of myofibroblasts and extensive angiogenesis. Consistent with the results of our study, postoperative inflammatory markers in their study were elevated; however, because of the different surgical methods, the inflammatory response was different. The inflammatory index was the lowest in TLDG.

Nutrition, immunity, inflammation and cancer are closely linked to, which may in turn affect the survival prognosis of cancer patients (9, 19). Gastric cancer patients often suffer from symptoms such as weight loss, hypoproteinaemia, anaemia and malabsorption, which are related to the inhibition of humoral and cellular immune functions, changes in inflammatory response and wound healing (20–23). A is used to reflect the nutritional status of the body; however, there are many influencing factors, such as the effect of general anaesthesia drugs on the liver, causing a decrease in protein. Changes in the expression levels of A may be important markers reflecting the prognosis of patients with gastric cancer (24). Therefore, this study used PNI to assess the nutritional status of patients. This is calculated using serum albumin and is an objective indicator of malnutrition, but A is the most widely used and the easiest to study. PNI was used to assess perioperative immunonutrition status and surgical risk in patients undergoing gastrointestinal surgery (25). Studies have shown that low PNI means poor immunonutrition, which may affect the immunity of the organism to the tumour and increase the burden of the tumour, thus affecting the overall prognosis of advanced cancer. Moreover, Jiang et al. (26) suggested that low PNI is associated with poor prognosis of malignant solid tumours and is included in routine nutritional assessment of patients with advanced gastric cancer (26–31). In radical distal gastrectomy, most of the stomach, including tumours and normal tissues, is removed, leading to malnutrition, which greatly increases the risk of tumour recurrence. Surgical trauma may inhibit the body's fluid and cellular immune function and stimulate the body to produce inflammation and traumatic changes, resulting in poor nutrient intake; therefore, different surgical methods lead to different degrees of decline in nutritional indicators, which is consistent with our findings. ΔPNI and ΔA were observed to have the least change in TLDG from preoperative to postoperative 1 day (Table 3), indicating that this procedure is to minimise the loss of nutrients in the body. Perioperative gastric cancer patients are beneficial to nutritional recovery and enhance immunity against tumour recurrence.

In addition, we studied the changes of inflammatory index and nutritional index of different surgical methods at 1 week postoperatively. NLR, N and WBC were found to be statistically significant. Although PLR was not statistically significant, TLDG showed the lowest inflammation in the index status. PNI and A were found to be statistically significant in the nutritional indicators. This makes us more convinced that TLDG has the weakest effect on the level of inflammatory response in the body and has the least impact on the nutritional status of the body.

Although this study yields the above meaningful results, there were some limitations to the current study. First, this is a retrospective study. Despite strict inclusion and exclusion criteria, certain selection biases remained. Second, although postoperative PLR levels were elevated, they were not statistically significant, probably due to sample size problems. Third, this study did not evaluate the prognosis, but we will further study the prognosis.

## Conclusions

The postoperative inflammatory response and nutritional status of patients with distal gastric cancer are related to surgery. TLDG has little effect on inflammatory response and nutritional status compared with LADG and ODG.

## Data Availability

The raw data supporting the conclusions of this article will be made available by the authors, without undue reservation.
